# A Potential Antifungal Effect of Chitosan Against *Candida albicans* Is Mediated via the Inhibition of SAGA Complex Component Expression and the Subsequent Alteration of Cell Surface Integrity

**DOI:** 10.3389/fmicb.2019.00602

**Published:** 2019-03-26

**Authors:** Pei-Yu Shih, Yu-Ting Liao, Yi-Kai Tseng, Fu-Sheng Deng, Ching-Hsuan Lin

**Affiliations:** Department of Biochemical Science and Technology, College of Life Science, National Taiwan University, Taipei, Taiwan

**Keywords:** chitosan, *Candida albicans*, SAGA complex, *ADA2*, cell wall

## Abstract

Due to the high incidence of nosocomial *Candida albicans* infection, the first-line drugs for *C. albicans* infection have been heavily used, and the emergence of drug-resistant strains has gradually increased. Thus, a new antifungal drug or therapeutic method is needed. Chitosan, a product of chitin deacetylation, is considered to be potentially therapeutic for fungal infections because of its excellent biocompatibility, biodegradability and low toxicity. The biocidal action of chitosan against *C. albicans* shows great commercial potential, but the exact mechanisms underlying its antimicrobial activity are unclear. To reveal these mechanisms, mutant library screening was performed. *ADA2* gene, which encodes a histone acetylation coactivator in the SAGA complex, was identified. Transmission electronic microscopy images showed that the surface of chitosan-treated *ada2*Δ cells was substantially disrupted and displayed an irregular morphology. Interestingly, the cell wall of *ada2*Δ cells was significantly thinner than that of wild-type cells, with a thickness similar to that seen in the chitosan-treated wild-type strain. Although *ADA2* is required for chitosan tolerance, expression of *ADA2* and several Ada2-mediated cell wall-related genes (*ALS2, PGA45*, and *ACE2*) and efflux transporter genes (*MDR1* and *CDR1*) were significantly inhibited by chitosan. Furthermore, *GCN5* encoding a SAGA complex catalytic subunit was inhibited by chitosan, and *gcn5*Δ cells exhibited phenotypes comparable to those of *ada2*Δ cells in response to chitosan and other cell surface-disrupting agents. This study demonstrated that a potential antifungal mechanism of chitosan against *C. albicans* operates by inhibiting SAGA complex gene expression, which decreases the protection of the cell surface against chitosan.

## Introduction

Due to the increase in the aging population and medical advances in the management of immunocompromised patients, the incidence of invasive fungal infections has risen dramatically (Cassone and Cauda, 2012; Dall et al., 2013; Papon et al., 2013). Among them, *Candida albicans* is the most predominant cause of fungal infections in humans. *C. albicans* is a commensal organism inhabiting multiple sites in humans (Martin, 1999; Pappas et al., 2004; Weiner et al., 2016). However, *C. albicans* can become pathogenic (Cassone and Cauda, 2012; Papon et al., 2013), and the infections occur primarily in immunocompromised patients (Papon et al., 2013; Kullberg and Arendrup, 2015). Without appropriate treatment, life-threatening sepsis caused by *C. albicans* infection can occur, with a crude mortality rate of up to 50% (Delaloye and Calandra, 2014).

Currently, the clinical treatment of fungal infections mainly depends on four classes of drugs (nucleoside analogs, azoles, echinocandins, and polyenes) (Robbins et al., 2016). Together, the limited choices and increasing global use of antifungal drugs can potentially result in resistance increases. During the last two decades, the emergence of human fungal pathogens has dramatically increased worldwide (Bertagnolio et al., 2004; Wisplinghoff et al., 2004; Yang et al., 2010), leading to a reduction in the efficacy of treatments for fungal infection (Martin, 1999; Angiolella et al., 2008; Chang et al., 2013; Ford et al., 2015). Thus, novel promising therapeutic strategies or new antifungal agents must be developed (Brown et al., 2012; Roemer and Krysan, 2014).

Chitosan [poly-(β-1→4)-2-amino-2-deoxy-D-glucopyranose] is a natural, biodegradable, and non-toxic linear polysaccharide derived from deacetylated chitin (Kong et al., 2010; Cheung et al., 2015). Chitosan has been widely used in many biomedical and agricultural applications and in the food, water treatment, and cosmetics industries (Shahidi et al., 1999; Kumar, 2000; Haque et al., 2005; Kim et al., 2005; Yamada et al., 2005; Azuma et al., 2015; Cheung et al., 2015). Furthermore, chitosan has been reported to have broad-spectrum antimicrobial activity against Gram-positive bacteria, Gram-negative bacteria and fungi (Kendra and Hadwiger, 1984; Hirano and Nagao, 1989; Tikhonov et al., 2006; Pena et al., 2013; Cheung et al., 2015). Several review articles have shown that the antimicrobial activity levels of chitosan are highly associated with its degree of deacetylation and pH (Kong et al., 2010; Cheung et al., 2015; Hosseinnejad and Jafari, 2016). In particular, a higher degree of deacetylation increases the antimicrobial activity of chitosan. Additionally, the antifungal and antimicrobial effects of chitosan are influenced by pH; higher antimicrobial activity is observed at lower pH values. Chitosan has been suggested to have antimicrobial activity as a cationic polymer when the pH is below 6.5 (Lim and Hudson, 2003; Rabea et al., 2003; Pena et al., 2013). Therefore, the positively charged chitosan can interact with the negatively charged microbial cell surface and disrupt the anion–cation balance, thereby exerting an inhibitory effect (Martinez-Camacho et al., 2010). Thus, the antimicrobial activity of chitosan depends greatly on its properties and on the type of bacteria or fungi involved (Kong et al., 2010; Cheung et al., 2015; Hosseinnejad and Jafari, 2016).

The biocidal action of chitosan against microorganisms shows great commercial potential, but the mechanisms underlying this antimicrobial activity remain largely unknown. The results of a previous study in which array profiling of the response of *Staphylococcus simulans* and *Staphylococcus aureus* to chitosan was analyzed suggest that the antibacterial activity of chitosan is possibly due to its binding to the cell surface, which leads to interference with bacterial energy metabolism and the electron transfer chain (Raafat et al., 2008). Furthermore, the genetic profiling of chitosan-treated *Saccharomyces cerevisiae* has been performed, and chitosan treatment was reported to result in three major transcriptional responses (Zakrzewska et al., 2005). These responses involved the stress-response factor Cin5p; Crz1p, which is involved in the calcineurin pathway; and the Rlm1p transcription factor, which is required for cell wall integrity (Zakrzewska et al., 2005). Furthermore, chitosan-treated *S. cerevisiae* was more resistant to the cell wall-degrading enzyme (CWDE) β-1,3-glucanase, suggesting that chitosan might be a plasma membrane-perturbing compound (Zakrzewska et al., 2005). These studies in bacteria and budding yeast implied that the maintenance of a functional cell membrane and cell surface are important in chitosan tolerance.

In this study, we first identified 38 transcriptional regulators and 11 cell wall-related genes involved in chitosan resistance through mutant library screening. Among these genes, *ADA2* and *CRZ1*, were selected for further analyses because *ada2*Δ exhibited the lowest optical density in the mutant library screening and is responsible for cell wall integrity (Bruno et al., 2006; Sellam et al., 2009) and because *CRZ1* in budding yeast has been shown to be required for chitosan resistance (Zakrzewska et al., 2005). However, although *crz1*Δ in SC5314 was showed to be highly sensitive to chitosan (unpublished data), expression of *CRZ1* did not exhibit a significant change in response to chitosan. Thus, this work mainly focuses the roles of Ada2 in *C. albicans* in response to chitosan.

The *ADA2* (alteration/deficiency in activation 2) gene was first identified in *S. cerevisiae* (Berger et al., 1992). *ADA2* is a component of the Spt-Ada-Gcn5-acetyltransferase (SAGA) complex, which plays an important role in histone acetylation and is involved in the regulation of numerous genes (Wang et al., 1998; Daniel and Grant, 2007). The capabilities of the SAGA complex factors for histone acetylation and interaction with acidic activation domains have been widely studied, and a core subunit of the SAGA Gcn5-Ada2-Ada3 complex is required to catalyze nucleosome acetylation (Marcus et al., 1994; Belotserkovskaya et al., 2000; Balasubramanian et al., 2002; Baker and Grant, 2007). Gcn5 exhibits histone acetyltransferase (HAT) activity and can acetylate N-terminal lysines on histones. In addition, the histone acetylation process on nucleosomes requires Ada3 (Ngg1) and Ada2 (Marcus et al., 1994; Balasubramanian et al., 2002). Null mutations in any SAGA complex component result in slow growth and lower resistance to environmental stress (Berger et al., 1992). For example, *ADA2* deletion could result in the instability of the SAGA complex, leading to a decrease in histone acetylation and the development of a stress sensitivity phenotype (Daniel and Grant, 2007).

*C. albicans* contains high-similarity homologs of the *S. cerevisiae* SAGA complex components (Sellam et al., 2009; Chang et al., 2015). The *ADA2* gene also plays an important role in histone acetylation in *C. albicans*; *ADA2* deletion strains showed reduced H3K9 acetylation (Sellam et al., 2009). Chromatin immunoprecipitation (ChIP) assays further verified that Ada2 is recruited and can bind to 200 gene promoters and that Ada2 is involved in mediating the expression of numerous genes, including genes related to glycolysis, pyruvate metabolism, oxidative stress, drug responses, and cell wall responses (Sellam et al., 2009). In particular, a cell wall stress response mediated by Rlm1, Ada2, and Cas5 has been proposed to be required for cell wall integrity (Bruno et al., 2006; Sellam et al., 2009). Furthermore, the protective effects of the ATP-binding cassette (*ABC*) and major facilitator superfamily (*MFS*) efflux systems directly regulated by Ada2 are required for tolerance to antifungal drugs (Sanglard et al., 2009; Ramirez-Zavala et al., 2014).

Many studies have shown that the antimicrobial effect of chitosan targets the cell surface, which suggests that chitosan is a good alternative approach for antifungal therapy (Kong et al., 2010; Pena et al., 2013; Cheung et al., 2015; Hosseinnejad and Jafari, 2016). Understanding more about the basic mechanisms will be extremely useful and will enable us to apply this understanding more specifically to the clinical treatment of fungal infections. In this work, we found that *ada2*Δ cells were highly sensitive to chitosan. Strong evidence that Ada2 is directly involved in cell surface composition and integrity was found by transmission electron microscopy (TEM). Additionally, instead of being induced, the expression of *ADA2, GCN5* and several Ada2-mediated cell wall-related genes was significantly repressed under chitosan challenge. Together, these findings indicate that a potential antifungal mechanism of chitosan operates by antagonizing SAGA complex gene expression, thereby altering the integrity of the *C. albicans* cell surface.

## Materials and Methods

### Media and Growth Conditions

Unless otherwise stated, all chemicals used in this study were purchased from Sigma-Aldrich Corporation (St. Louis, MO, United States). Luria-Bertani (LB) medium and yeast extract-peptone-dextrose (YPD) medium were prepared as previously described (Liang et al., 2014). YPD agar supplemented with 0.2 mg/ml nourseothricin (NAT, Werner BioAgents, Jena, Germany) was used to select and maintain NAT-resistant *C. albicans* strains. Growth curves were measured with a Biowave density meter as previously described (Deng and Lin, 2018). YPD agar supplemented with 0.04% SDS was used in the spot dilution assay. RPMI1640 liquid medium was prepared from 0.165 M morpholinepropanesulfonic acid (MOPS) and 2% glucose and was adjusted to a pH of 7.0 for the medium of the control group, as previously described (Alburquenque et al., 2010; Chien et al., 2013). The medium for the buffer control group was prepared from RPMI 1640 medium supplemented with 0.2% acetic acid, with a final pH of 6.3. The chitosan medium was prepared from RPMI 1640 supplemented with 0.2% chitosan dissolved in 0.2% acetic acid (experimental group). The chitosan used in this study was purchased from Shin Era Technology (Taipei, Taiwan). The MW of chitosan is approximately 23 kDa (20–30 kDa), and the degree of deacetylation of chitosan is approximately 94% (Tsai et al., 2011; Chien et al., 2013). To prepare RPMI 1640 agar, a double concentration of RPMI 1640 medium was prepared from 0.3 M MOPS and 4% glucose. After sterilization through a 0.22-μm membrane filter (GeneDireX, Las Vegas, NV, United States), the double concentration of RPMI 1640 medium was mixed with an equal volume of sterilized 3% agar. RPMI 1640 medium supplemented with a final concentration of 0.2% acetic acid or 0.2% chitosan was mixed with the sterilized agar.

### Mutant Library Screening

The *C. albicans* mutant library derived from BWP17 was purchased from the FGSC website^1^ (Davis et al., 2002; Nobile and Mitchell, 2005; Richard et al., 2005; Norice et al., 2007; Rauceo et al., 2008). To obtain a relatively optimal condition for the screening, three different concentrations (0.1%, 0.2%, and 0.5%) of chitosan dissolved in 0.1%, 0.2%, and 0.5% acetic acid, respectively, were tested. The *C. albicans* wild-type strain was first culture in the RPMI, buffer control or RPMI + chitosan for 24 or 48 h and spotted on the chitosan free YPD plates after washed with PBS twice (Supplementary Figure S1). Each *C. albicans* transcription factor and cell surface mutant strain was separately grown in YPD liquid medium in 96-well microplates at 30°C overnight. Strains were then transferred into 96-well microplates containing RPMI 1640 liquid medium (supplemented with 0.165 M MOPS and 2% glucose). The buffer control group in the mutant library screen was prepared from RPMI 1640 medium supplemented with 0.5% acetic acid. The chitosan medium was prepared from RPMI 1640 supplemented with 0.5% chitosan dissolved in 0.5% acetic acid (experimental medium). Microplates were incubated at 30°C for 48 h, and the optical density was then measured at 600 nm in a SpectraMax 190 microplate reader (Molecular Devices, San Jose, CA, United States). The absorbance value for each mutant treated with chitosan or acetic acid was compared to determine the susceptibility of the strains to chitosan, and statistical significance was determined by Student’s *t*-test.

### Plasmid and Strain Construction

The *C. albicans* strains and primers used in this study are listed and described in Supplementary Tables S1, S2, respectively. To delete the *ADA2* gene, two PCR cycles were performed. The 5′ and 3′ flanking regions of *ADA2* were amplified using the primer sets 1021/1022 and 1147/1148, respectively. These PCR products were digested with *Apa*I/*Xho*I and *Not*I/*Sac*Π, respectively, and were inserted into the pSFS2A plasmid (Reuss et al., 2004) to generate the pSFS-*ADA2*KO plasmid. This construct was linearized by *Apa*I/*Sac*II digestion and was transformed into strain SC5314 (YL2) to generate heterozygous *ada2Δ/ADA2* strains. The *SAT1* marker was recycled by treatment with 2% maltose. The heterozygous strains were retransformed with the same deletion construct to generate the *ada2*Δ strains (YL1693 and YL1694). Mutants were verified with the primer sets 1019/1020, 6/1017 and 7/1018 (Supplementary Figure S2). To generate the *ADA2* complemented strains, the coding region of the *ADA2* gene and ∼1.5 kb of upstream sequences obtained from http://www.candidagenome.org/ were amplified from the SC5314 genome by PCR with the primer set 1149/1150. The PCR products were then digested with *Kpn*I/*Apa*I and inserted into the pSFS2A plasmid to generate the pSFS-*ADA2*AB plasmid. These constructs were linearized by *BmgB*I digestion and transformed into *ada2*Δ to generate the *ada2Δ::ADA2* strains (YL1687 and YL1689). The *ADA2* complemented strains were confirmed with the primer set 1019/1020. To delete *GCN5*, the 5′ and 3′ flanking regions of *GCN5* were amplified using the primer sets 1251/1252 and 1253/1254, respectively. The PCR products were digested with *Apa*I/*Xho*I and *Sac*II/*Sac*I and cloned into pSFS2A to generate pSFS-GCN5 KO. This plasmid was then digested with *Apa*I/*Sac*I and transformed into strain SC5314 to generate heterozygous *gcn5Δ/GCN5* strains. The *SAT1* marker was recycled by treatment with 2% maltose. The heterozygous strains were retransformed with the same deletion construct to generate the *gcn5*Δ strains (YL1789 and YL1790). Primers 1255/1256, 6/1257 and 7/1258 were used to verify the *gcn5*Δ genotype. To construct the functional *GCN5* complemented strains, the *GCN5* coding region and ∼0.7 kb of upstream sequences were amplified using the primer set 1347/1348. The PCR products were digested with *Apa*I/*Xho*I and inserted into the pSFS2A-GCN5 KO plasmid to generate the pSFS-*GCN5*AB plasmid. These constructs were linearized with *Apa*I/*Sac*I and transformed into the *gcn5*Δ strains to generate the *gcn5Δ::GCN52* strains (YL1828 and YL1829). The *GCN5* complemented strains were confirmed with the primer set 1255/1256.

### Sensitivity Assays

Overnight cultures of *C. albicans* cells at a OD_600_ of 1.0 were diluted by 10-fold serial dilutions. Each dilution of 5 μl (from 1 × 10^8^ to 1 × 10^4^ cells) was spotted onto RPMI 1640 agar, RPMI 1640 agar containing 0.2% acetic acid (buffer control group), or RPMI 1640 agar supplemented with 0.2% chitosan (experimental group). To test the cell wall-disrupting agents, final concentrations of 60 μM calcofluor white and 0.2 μg/ml caspofungin were used (Zakrzewska et al., 2005; Wang et al., 2011; Delgado-Silva et al., 2014). The cell membrane sensitivity test was conducted on agar with a final concentration of 0.04% SDS (Wang et al., 2011). Plates were incubated at 30°C for 2 days, and images were obtained.

### Quantitative Reverse Transcription Polymerase Chain Reaction

The assay was followed the established protocol in our laboratory (Chang et al., 2016). A total of 200 μl of *C. albicans* cells cultured overnight were transferred into 10 ml of fresh YPD liquid. Cells were collected by centrifugation at 3,000 rpm for 10 min and were washed with sterile water three times. Cells of each sample were treated with RPMI 1640 medium, RPMI 1640 medium containing 0.2% acetic acid (buffer control group), or RPMI 1640 medium supplemented with 0.2% chitosan (experimental group) at 30°C for 20 min. Cells were harvested by centrifugation at 3,000 rpm for 10 min and were washed with sterile water three times. Total RNA was extracted from the cells using a MasterPure^TM^ Yeast RNA Purification Kit (Epicentre, Madison, WI, United States), and DNA was removed with DNase I (Thermo Fisher Scientific, Waltham, MA, United States). RNA was transcribed to complementary DNA (cDNA) with an iScript^TM^ cDNA Synthesis Kit (Bio-Rad Laboratories., Inc., Hercules, CA, United States). Quantitative PCR was performed on a Bio-Rad CFX Manager (Bio-Rad Laboratories, Hercules, CA, United States). Each experiment was independently repeated three times, and the means of the triplicates are shown. The differences between the control group and the experimental group were analyzed with Student’s *t*-test. The primer sets 541/542, 1085/1086, 1228/1229, 1234/1235, 1238/1239, 1087/1088,1089/1090, 1493/1494, 1558/1559 and 1560/1561 were used for the detection of *ACT1, ADA2, ALS2, PGA45, ACE2, MDR1, CDR1* and *GCN5* expressions, respectively. Student’s *t*-test was used for statistical analyses. The expression of the target genes was normalized to the expression of the *ACT1* gene. The Cq values of *ACT1* gene were stable in each biological experiment (Supplementary Table S3).

### Transmission Electron Microscopy

The wild-type and *ada2*Δ strains treated with RPMI 1640 medium, RPMI 1640 medium containing 0.2% acetic acid, or RPMI 1640 medium supplemented with 0.2% chitosan at 30°C for 20 min were washed with sterile water three times. The washed samples were fixed with 2.5% glutaraldehyde at room temperature for 80 min and were then washed with 0.1 M phosphate buffer three times for 15 min each. Samples were post-fixed with 1% osmium tetroxide (OsO_4_) at room temperature for 1 h and were washed with 0.1 M phosphate buffer three times for 15 min each. Subsequently, the samples were dehydrated by immersion in a graded ethanol series (30%, 50%, 70%, 90%, and 100%) for 15 min and were further dehydrated by immersion in 100% acetone two times for 30 min each. Finally, the samples were processed with different ratios of 100% acetone:Spurr’s resin for 4 h and with pure Spurr’s resin (Electron Microscopy Science, Hatfield, PA, United States) overnight. The samples were embedded in pure Spurr’s resin at 70°C for 2 days (Rico et al., 1991; Vazquez-Munoz et al., 2014). The blocks of samples were sliced into thin sections by an ultramicrotome to produce sections of 50–70 nm thickness and were placed on a copper grid for observation with TEM. For cell wall thickness measurement, images were obtained at 8,000×, 30,000×, and 50,000× magnification. The measurements of the cell wall thickness in *C. albicans* cells were quantitated by using DigitalMicrograph software.

## Results

### Mutant Library Screening Determined the Involvement of *ADA2* in *C. albicans* Chitosan Resistance

**FIGURE 1:**
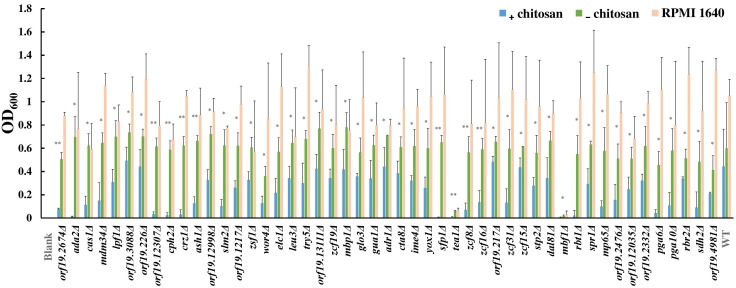
Results of chitosan treatment screening of the transcription factor and cell surface-related gene knockout library. Each mutant strain was separately grown in RPMI 1640 liquid medium (RPMI), RPMI containing 0.5% acetic acid (RPMI + buffer) or RPMI containing both 0.5% acetic acid and chitosan (RPMI + buffer + 0.5% chitosan) in 96-well microplates. The microplates were incubated at 37°C for 48 h, and the optical density of the cells was measured at 600 nm. The absorbance of each mutant strain treated with chitosan or acetic acid was compared to determine the susceptibility of the strains to chitosan. Statistical significance was determined by Student’s *t*-test (unpaired, two-tailed). ^∗^*P* < 0.05; ^∗∗^*P* < 0.01.

To understand the mode of action of chitosan against *C. albicans*, mutant library screening was performed. A total of 337 transcription factor mutant strains and 186 cell surface-related gene mutant strains were tested with or without chitosan treatment (Supplementary Table S4). A total of 38 transcription factor gene mutant strains and 11 cell surface-related gene mutant strains exhibited a significant reduction in cell growth after chitosan treatment (Figure 1 and Supplementary Table S4). Functional analyses of these potential chitosan-response genes revealed diverse functions, including involvement in biofilm formation (17 genes), cell adhesion (8 genes), hyphal formation (5 genes), virulence (2 genes) and antifungal-related responses (9 genes). In addition, the function of 13 genes remains unclear (Supplementary Table S4), although *ORF19.2476, ORF19.2332* and *ORF19.4981* were characterized as cell wall-related genes in the mutant library (Davis et al., 2002; Nobile and Mitchell, 2005; Richard et al., 2005; Norice et al., 2007; Rauceo et al., 2008). Ada2 was selected for further investigation because the lowest optical density was observed for the *ADA2* mutant (Figure 1 and Supplementary Table S4).

### *ADA2* Gene Deletion Resulted in High Chitosan Susceptibility in *C. albicans* SC5314

**FIGURE 2:**
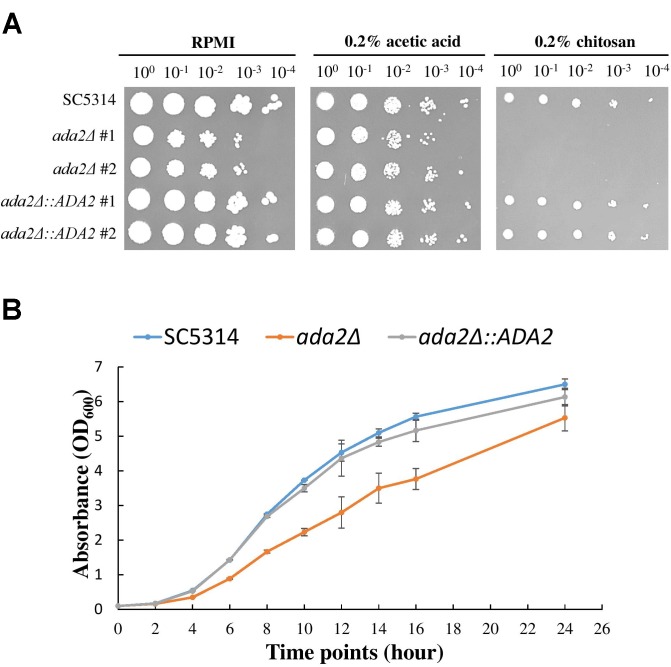
*C. albicans ada2*Δ strains were highly sensitive to challenge with chitosan. **(A)** The deletion of *ADA2* resulted in a mild growth defect in the RPMI and buffer control groups (0.2% acetic acid) and completely abolished growth in 0.2% chitosan. Growth and chitosan resistance were rescued in the revertant strains. **(B)** Overnight cultures of *C. albicans* cells were diluted to an OD_600_ of 0.1 in fresh YPD liquid medium. Growth rates were monitored with a Biowave density meter. The experiments were performed in biological triplicate.

To further confirm the screening results, *ada2*Δ strains and complemented strains were constructed from the standard *C. albicans* SC5314 strain and were tested for sensitivity to chitosan. As shown in Figure 2A, the *ada2*Δ strains in the RPMI and 0.2% acetic acid buffer control groups exhibited a mild growth defect, while the growth of the mutants was completely abrogated on medium supplemented with 0.2% chitosan. The reintroduction of a functional copy of *ADA2* into the *ada2*Δ strains rescued the chitosan resistance. These results indicate that *ADA2* is necessary for the adaptation of *C. albicans* to chitosan. Furthermore, the growth curves of the wild-type, *ada2*Δ and complemented strains showed that *ADA2* deletion could cause a mild growth defect (Figure 2B).

### Chitosan Damaged the *C. albicans* Cell Wall and Cell Membrane, and This Effect Was Accentuated in *ada2*Δ Cells

**FIGURE 3:**
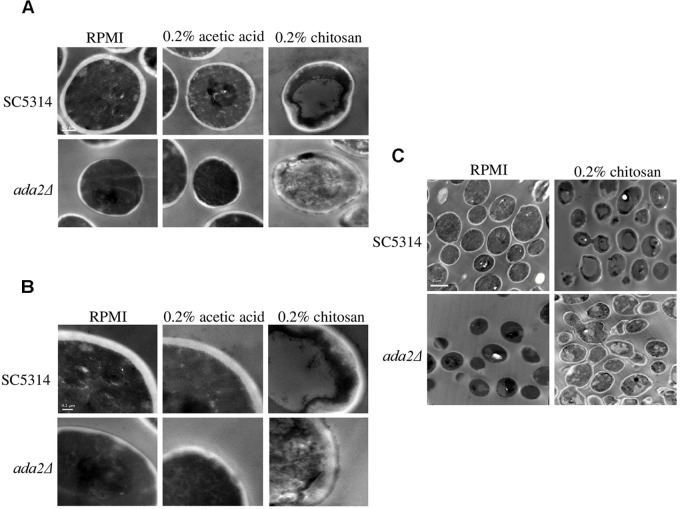
Chitosan treatment disrupted the integrity of the *C. albicans* cell wall and cell membrane, and this effect was accentuated in *ada2*Δ cells. Images were obtained at **(A)** 30,000×, **(B)** 50,000×, and **(C)** 8,000× magnifications. Cells of the wild-type SC5314 strain showed well-defined boundaries in either RPMI or in RPMI + 0.2% acetic acid (buffer control medium) but exhibited slightly irregular cell morphologies after chitosan treatment. The deletion of the *ADA2* gene resulted in ill-defined cell surfaces in both the RPMI and buffer control groups, whereas chitosan-treated *ada2*Δ cells exhibited a collapsing morphology. Scale bars of 0.5 μm, 0.2 μm, and 2 μm are shown on the representative images obtained at 30,000×, 50,000×, and 8,000× magnification, respectively.

TEM showed that neither RPMI nor RPMI containing 0.2% acetic acid caused obvious effects on wild-type cells; while the cell surface of *ada2*Δ cells were aberrant in both conditions (Figure 3). However, treatment with 0.2% chitosan for 20 min resulted in the disruption of cell surface integrity in *C. albicans* SC5314 cells, which sporadically displayed aberrant morphologies (Figure 3). The morphology of *ada2*Δ cells was even more severely affected; the boundary between the cell wall and cell membrane was ambiguous, and the cell wall structural integrity was indefinite (Figure 3).

### Cell Wall Thickness Was Reduced in Both the *ada2*Δ Cells and the Chitosan-Treated SC5314 Cells

**FIGURE 4:**
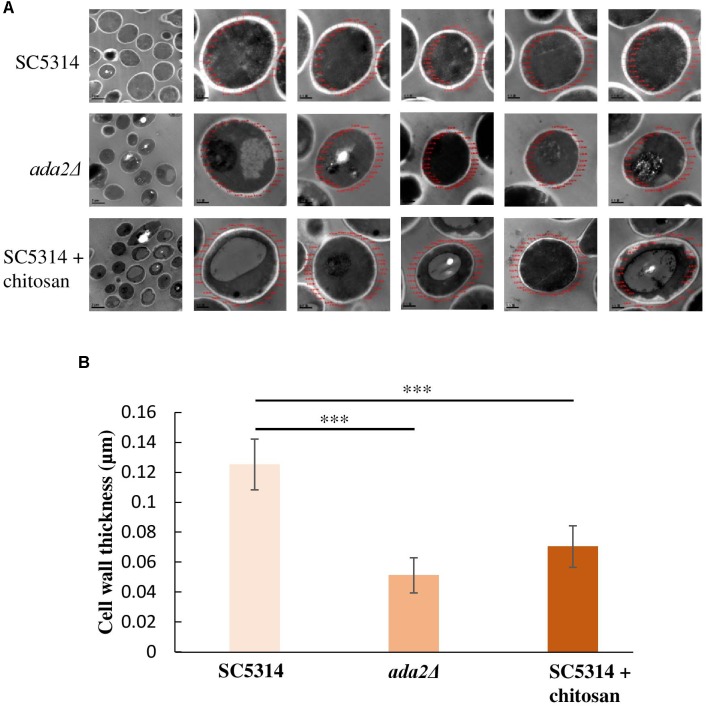
Cell wall thickness was significantly reduced in both the *ada2*Δ strain and the chitosan-treated wild-type SC5314 strain. **(A)** Five individual cells from TEM images obtained at 8,000× magnification were randomly selected, and their cell wall thickness was measured at 30,000× magnification using DigitalMicrograph software. **(B)** Thirty sites around the circumference of each selected cell were measured. The cell wall thickness of *ada2*Δ cells was significantly lower than that of wild-type SC5314 cells. Chitosan-treated wild-type SC5314 cells showed a statistically significant reduction in cell wall thickness comparable to that seen in *ada2*Δ cells. The values are the means ± SDs of at least three experimental replicates. ^∗∗∗^*P* < 0.001 compared with the value for untreated wild-type SC5314 cells. Statistical significance was determined using Student’s *t*-test.

Unexpectedly, even without chitosan treatment, *ADA2* deletion strains exhibited an extremely thin cell wall (Figure 3C). This phenomenon was also observed in the chitosan-treated wild-type strain (Figure 3C). To determine the cell wall thickness, five individual cells of the SC5314 wild-type, *ada2*Δ and chitosan-treated wild-type strains were selected. Thirty sites around the circumference of each cell were measured to quantitate the cell wall thicknesses (Figure 4A). The quantitative results showed that the untreated wild-type strain exhibited a cell wall thickness of 0.125 μm as measured between the plasma membrane and the cell wall, while the cell wall thickness in the *ada2*Δ and chitosan-treated wild-type strains was significantly lower, ranging from 0.04 to 0.06 μm and 0.06 to 0.07 μm, respectively (Figure 4B). These data provide direct evidence supporting the results of several previous studies (Bruno et al., 2006; Sellam et al., 2009) indicating that the existence of Ada2 controls the cell wall damage response. Furthermore, based on these findings, we proposed that a potential antifungal mechanism of chitosan against this fungus operates by antagonizing *ADA2* expression, thereby altering the integrity of the *C. albicans* cell surface.

### The Thinner Cell Wall of *ada2*Δ Increased the Susceptibility to Cell Surface Stress-Inducing Agents

**FIGURE 5:**
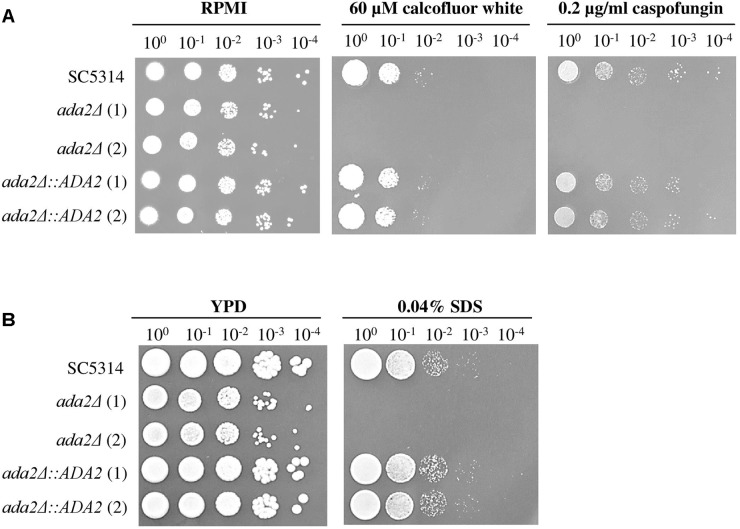
*ada2*Δ cells were more susceptible to cell surface-disrupting agents than were wild-type cells. The growth of *ada2*Δ cells was completely abolished in the presence of **(A)** the cell wall-disrupting agents calcofluor white and caspofungin, and **(B)** the cell membrane-disrupting agent SDS. The wild-type SC5314 and complemented strains exhibited similar growth.

The *C. albicans* Ada2 protein is required for resistance to cell stressors, including fluconazole, caspofungin and oxidative stress (Bruno et al., 2006; Sellam et al., 2009; Ramirez-Zavala et al., 2014). Two cell wall-disturbing (calcofluor white and caspofungin) agents and one cell membrane-disturbing (SDS) agent were therefore chosen for use in sensitivity assays (Roncero and Duran, 1985; Ram et al., 1994). As shown in Figure 5A, compared to the growth of the wild-type strains, the growth of *ada2*Δ strains was completely abolished on medium supplemented with 60 μM calcofluor white, 0.2 μg/ml caspofungin or 0.04% SDS, whereas these stressors had only a mild effect on the growth of the wild-type strain (Figure 5). The *ADA2* complemented strains regained resistance to these cell surface stress agents (Figure 5).

### Chitosan Inhibited *ADA2* Gene Expression

**FIGURE 6:**
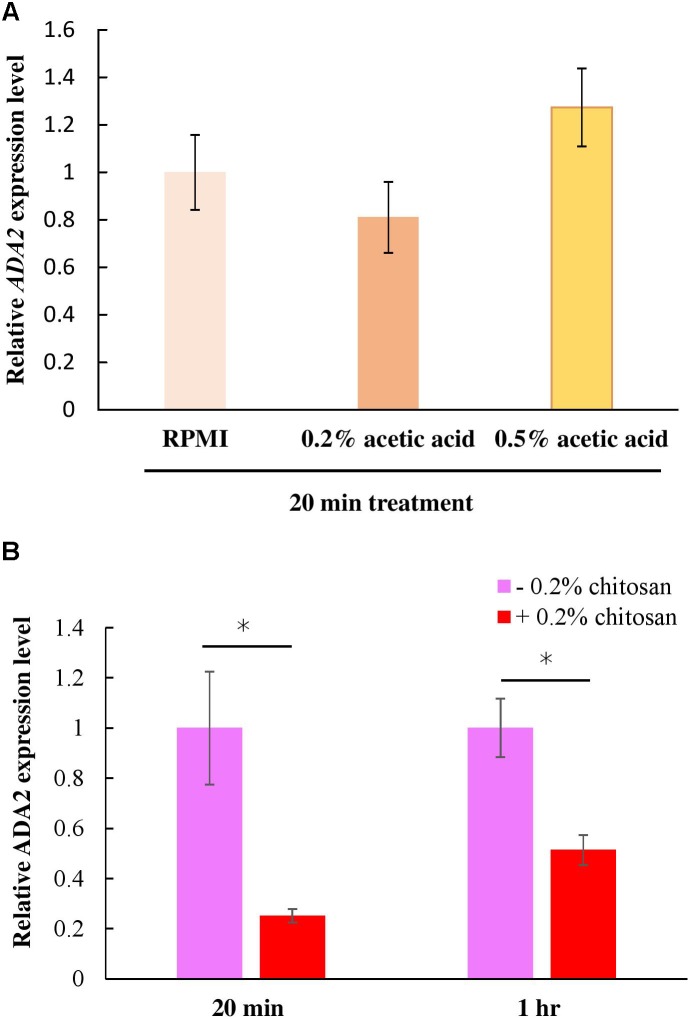
Chitosan, but not RPMI alone or buffer control (acetic acid), repressed *ADA2* gene expression. **(A)** Treatment with 0.2% or 0.5% acetic acid for 20 min did not affect *ADA* expression significantly, whereas **(B)** chitosan treatment of SC5314 cells for 20 min or for 1 h exhibited similar tendencies to dramatically reduce *ADA2* gene expression. The values are the means ± SDs of at least three experimental replicates. ^∗^*P* < 0.05 compared with the value for untreated wild-type SC5314 cells. *ADA2 e*xpression was normalized to that of the *ACT1* gene as indicated. Statistical significance was determined using Student’s *t*-test.

The *ada2*Δ cells and the chitosan-treated wild-type cells displayed identical phenotypes in terms of their thinner cell walls (Figure 4). This finding implies that chitosan or acetic acid, instead of inducing *ADA2* expression, might repress *ADA2*. To understand the effect of chitosan on *ADA2* expression, a quantitative polymerase chain reaction (PCR) analysis was performed. We first demonstrated that acetic acid was not an exogenous factor affecting *ADA2* expression; no significant difference in *ADA2* expression was found between cells cultured in RPMI and cells cultured in the acetic acid solution (Figure 6A). However, *ADA2* expression was significantly reduced in the wild-type strain after exposure to 0.2% chitosan for 20 min and for 1 h, compared with that in untreated *C. albicans* cells (Figure 6B).

### Cell Wall-Related Genes Positively Regulated by Ada2 Were Inhibited by Chitosan

**FIGURE 7:**
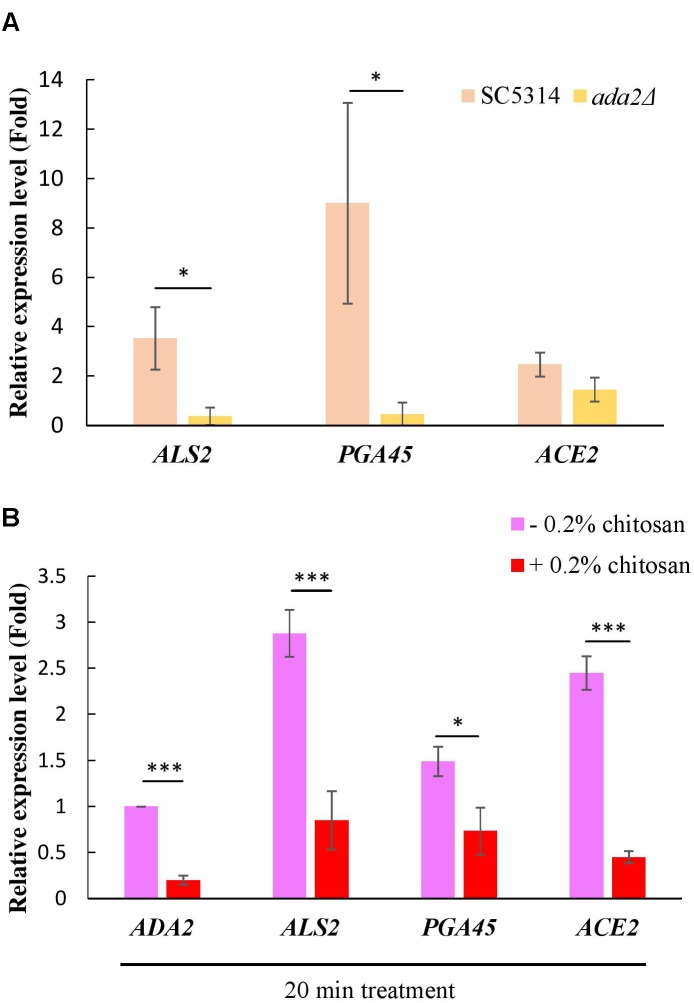
Expression of cell wall-related genes was positively regulated by Ada2 but negatively regulated by chitosan. **(A)** The expression of *ALS2, PGA45*, and *ACE2*, particularly *ALS2* and *PGA45*, was regulated by Ada2; *ADA2* deletion resulted in a significant reduction in the expression of these genes. A very slight, non-significant reduction in *ACE2* expression occurred. ^∗^*P* < 0.05 compared to the value for untreated wild-type SC5314 cells. **(B)** Chitosan treatment significantly reduced the expression of *ADA2, ALS2, PGA45*, and *ACE2* in wild-type SC5314 cells. The expression values were compared between the wild-type strain groups treated with and without chitosan. ^∗^*P* < 0.05, ^∗∗∗^*P* < 0.001, respectively. The expression values were normalized to the expression of the *ACT1* gene as indicated. Statistical significance was determined using Student’s *t*-test. The values are the means ± SDs of at least three experimental replicates.

A ChIP experiment demonstrated that many cell wall-associated genes are directly regulated by Ada2 (Sellam et al., 2009). Three genes, namely, *ALS2, PGA45*, and *ACE2*, were selected from this database (Sellam et al., 2009). Als2 is a putative GPI-anchor that belongs to the *ALS* family and plays a role in adhesion and biofilm formation (Hoyer et al., 1998; Hoyer, 2001). *PGA45* encodes a putative GPI-anchor cell wall protein (De Groot et al., 2003). Ace*2* is a cell wall transcription factor that regulates several cell wall-related genes, such as *ASH1, PIR1, PRY2*, and *RME1* (Kelly et al., 2004). In further support of the results of the previous study (Sellam et al., 2009), we found that the expression of these three genes, particularly *ALS2* and *PGA45*, was positively regulated by Ada2; *ADA2* deletion resulted in a significant reduction in the expression of these genes (Figure 7A). A slight but non-significant reduction in *ACE2* expression occurred (Figure 7A). Additionally, treatment of the *C. albicans* wild-type strain with chitosan resulted in a significantly reduction in the expression of *ALS2, PGA45*, and *ACE2* (Figure 7B). These data firmly support the hypothesis that the molecular mechanism of chitosan operates by inhibiting *ADA2* in *C. albicans* in order to repress the expression of several cell surface-related genes, thus affecting the cell surface and reducing cell wall thickness.

### Efflux Pump Genes Regulated by Ada2 Were Repressed by Chitosan

**FIGURE 8:**
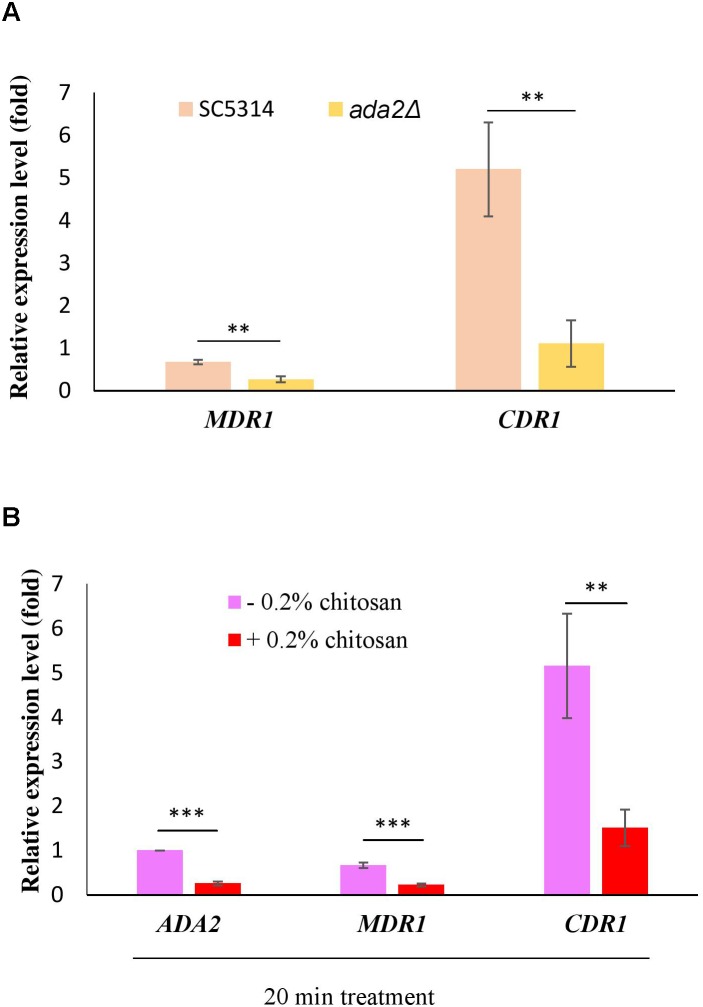
*C. albicans* Ada2-regulated efflux pump genes were repressed by chitosan. **(A)** The expression of both *MDR1* and *CDR1* is positively regulated by Ada2, as *ADA2* deletion significantly reduced the expression of *MDR1* and *CDR1*. ^∗∗^*P* < 0.01 compared to the value for untreated wild-type SC5314 cells. **(B)** In response to chitosan, the expression of *MDR1* and *CDR1* was significantly repressed. The expression values were compared between the wild-type strain groups treated with and without chitosan. ^∗∗^*P* < 0.01 and ^∗∗∗^*P* < 0.001, respectively. The expression values were normalized to the expression of the *ACT1* gene as indicated. Statistical significance was determined using Student’s *t*-test. The values are the means ± SDs of at least three experimental replicates.

Several reports have shown that the expression of the multidrug transporter genes *CDR1, CDR2* and *MDR1* can be induced by antifungal drugs and that these genes are associated with many clinical drug-resistant *C. albicans* strains (Goffeau et al., 1997; Kohli et al., 2001; Shukla et al., 2003; Pasrija et al., 2008). The direct regulation of *CDR1* and *MDR1* by Ada2 has been proved (Sellam et al., 2009). Consistent with the previous report (Sellam et al., 2009), *ADA2* deletion caused a significant reduction in the expression of both *MDR1* and *CDR1* (Figure 8A). However, the expression of *MDR1* and *CDR1* was significantly repressed after chitosan treatment (Figure 8B). These data reconfirm the finding that *C. albicans ADA2* is repressed by chitosan, leading to an decrease in the expression of Ada2-regulated genes regulating the *MDR1* and *CDR1* efflux pumps.

### The Gcn5 Histone Acetyltransferase Was Required for Chitosan Tolerance

**FIGURE 9:**
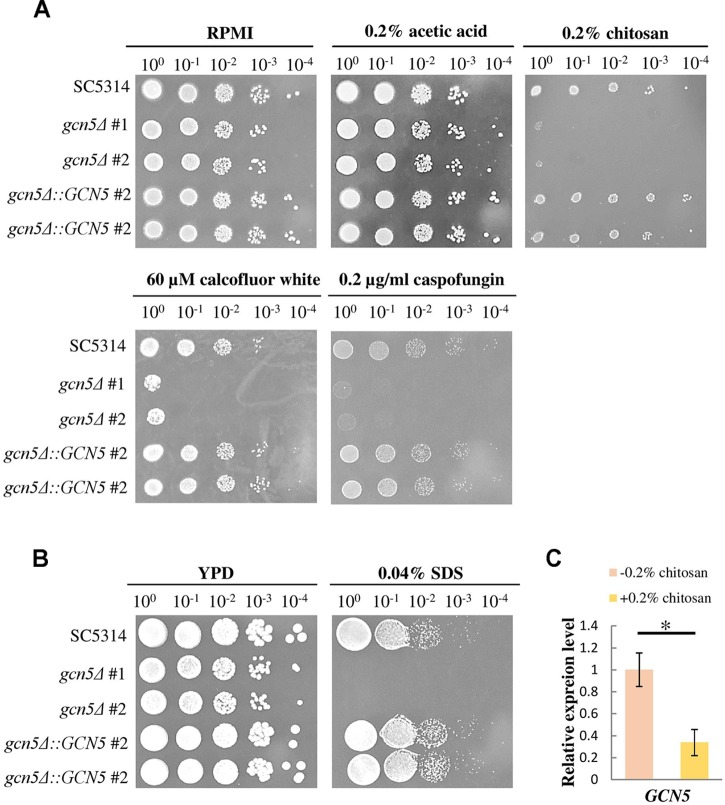
The *GCN5* histone acetyltransferase gene is required for resistance to chitosan and cell surface-disrupting agents, and *GCN5* expression is inhibited by chitosan. **(A)** *gcn5*Δ strains were highly sensitive to chitosan. Additionally, the growth of *gcn5*Δ cells was significantly inhibited by the cell wall stress agents calcofluor white and caspofungin and by **(B)** the cell membrane-disturbing agent SDS. The wild-type SC5314 strain and the complemented strain exhibited similar growth. **(C)** Chitosan treatment of SC5314 cells for 20 min significantly repressed *GCN5* expression. The values are the means ± SDs of at least three experimental replicates. ^∗^*P* < 0.05 compared with the value for untreated wild-type SC5314 cells. The expression values were normalized to the expression of the *ACT1* gene as indicated. Statistical significance was determined using Student’s *t*-test.

To confirm whether the repression of histone acetylation is associated with the mechanism underlying the inhibitory action of chitosan, mutants were constructed, and *GCN5* expression was tested. When treated with chitosan, calcofluor white, caspofungin and SDS, the *GCN5* null mutation strains showed phenotypes comparable to those of *ada2*Δ strains (Figure 9A,B). The complemented strains regained chitosan resistance. Furthermore, *GCN5* expression was significantly inhibited by chitosan (Figure 9C). These results indicated that chitosan represses the histone acetylation activity mediated by the SAGA complex in *C. albicans* and therefore inhibits fungal growth.

## Discussion

The antimicrobial activity of chitosan has been documented, but the mechanisms underlying its antifungal action on *C. albicans* remain unclear (Raafat et al., 2008; Kong et al., 2010; Pena et al., 2013; Cheung et al., 2015). Chitosan is believed to potentially interact with the cell surface, thereby altering cell permeability and blocking transport systems (Rabea et al., 2003; Kong et al., 2010). Interestingly, a few reports proposed that low molecular weight chitosan is able to penetrate the cell wall and interact with DNA, thereby inhibiting transcription (Hadwiger et al., 1986; Sudarshan et al., 1992). In this study, we identified 49 genes involved in chitosan resistance through a mutant library screening system, and most of these genes are currently under investigation. In particular, the involvement of these genes in adherence and antifungal-related responses is reasonable (Supplementary Table S4), given that chitosan can interact with the cell surface and that chitosan itself is a stressor that might be associated with certain antifungal responses. Interestingly, several genes required for chitosan resistance possess virulence-associated functions, including filamentation, biofilm development and virulence (Supplementary Table S4). The screening results implied that the chitosan used in this study not only had physical extracellular interaction abilities but also had a potential intracellular action. Notably, a few fungal pathogens, such as *Cryptococcus neoformans*, utilize chitosan to maintain cell wall integrity during the vegetative stage (Baker et al., 2007). Whether *C. albicans* can misrecognize chitosan as chitin and incorporate it into the cell wall, leading to reduced cell growth, remains an open question. In addition, *crz1*Δ in SC5314 was constructed and tested its susceptibility to chitosan. *crz1*Δ strains are required to survive in a chitosan environment, but *CRZ1* expression showed non-significant difference (unpublished data). The calcineurin-Crz1 pathway involved in stress resistance and can be activated several external stimuli, particularly Ca^2+^ (Rusnak and Mertz, 2000). The increase intracellular calcium leads to activation of the signal cascade (Cyert, 2003). The activated calcineurin then dephosphorylates Crz1p and promote the dephosphorylated (activated) form of Crz1 to move from cytosol into nucleus (Stathopoulos-Gerontides et al., 1999). It is possible that the chitosan effect on Crz1 mainly depends on post-translational *CRZ1* or *CRZ1* deletion causes a general effect on stress response. Additionally, whether the calcineurin cascade components, such as *CCH1, MID1, CNA1, CNB1*, also exhibit sensitive phenotypes to chitosan required further investigation.

The Ada2-Ada3-Gcn5 SAGA complex in budding yeast and *C. albicans* is sufficient for robust HAT activity, leading to either the induction or repression of the expression of certain genes (Naar et al., 2001; Daniel and Grant, 2007). Indeed, the *C. albicans* Ada2 targets a broad range of promoters and plays a global role in transcriptional regulation (Sellam et al., 2009). However, no potential Ada2-binding candidate gene required for chitosan tolerance was found in the mutant library screening data (Supplementary Table S4). Furthermore, three specific transcription factors, namely, Gal4, Cap1 and Mrr1, can be recruited by Ada2 to influence glycolysis, the oxidative stress response and drug resistance, respectively (Sellam et al., 2009). Despite observing a mild, non-significant growth defect in both the *gal4*Δ and *cap1*Δ strains in response to challenge with chitosan (Supplementary Table S4), we cannot eliminate the possibility that chitosan might have roles in controlling cell metabolism and could potentially elicit an oxidative stress response during the treatment. Furthermore, our results suggest that the drug resistance-related genes *CDR1* and *MDR1* might not have a role in chitosan resistance, given that Mrr1 controls *MDR1* expression and that the drug resistance observed in *C. albicans* and *mrr1*Δ strains in the screening data did not inhibit the response to chitosan (Supplementary Table S4) (Morschhauser et al., 2007). Under challenge with stressors, the Ada2-Ada3-Gcn5 SAGA complex coordinates with other cellular factors that modulate histone modification and gene regulation (Bruno et al., 2006; Sellam et al., 2009; Ramirez-Zavala et al., 2014) in order to adapt to different stimuli and host niches (Bruno et al., 2006; Sellam et al., 2009; Ramirez-Zavala et al., 2014). We found that the loss of *ADA2* in *C. albicans* resulted in a reduction of cell wall thickness of approximately 50%, which may explain why *ada2*Δ strains cannot survive in many unfavorable conditions and display weak virulence (Sellam et al., 2009). Bruno et al. (2006) have proposed that the transcription factor Cas5, which mediates the integrity of the *C. albicans* cell wall in response to caspofungin, could be recruited by the Ada2 protein. We presume that Ada2-Cas5 co-activation might also play an important role in chitosan adaptation in *C. albicans*.

We proposed that the inhibition of SAGA complex expression by chitosan is one of the biocidal mechanisms of chitosan against *C. albicans*. This hypothesis was further supported by the finding that *ADA2* and Ada2-regulated cell wall-related genes were inhibited by chitosan. Furthermore, the *GCN5* HAT is repressed in response to chitosan, and the *GCN5* mutant strains are also highly susceptible to chitosan, cell wall-disrupting (calcofluor white and caspofungin) and cell membrane-disrupting (SDS) agents. The data imply that chitosan treatment of *C. albicans* cells decreases the levels of chitin and β-glucan or alters the ultrastructure of the cell wall and cell membrane via the inhibition of SAGA complex component expression. Thus, analysis of the cell wall composition and organization of both chitosan treated WT and *ada2*Δ cells can provide a direct evidence to support our assumption. However, a major issue for performing the experiment is that chitosan-treated *C. albicans* cells were dramatically aggregated because the positively charged chitosan can interact with the negatively charged microbial cell surface. We are still looking a good way to solve this issue. Furthermore, it is also possible that the outcome results from the sum of indirect effects, in which a number of signaling pathways are required for chitosan response, leading to reduce cell wall integrity and alter gene expression or the traces of chitin present in the sample could also contribute to the impact. Finally, the mode of action of chitosan is highly dependent on the type of microorganism and its molecular weight and characteristic (Verlee et al., 2016).

The fungal cell wall, which is the interface between cells and the environment, provides mechanical strength and serves as a physical barrier to protect fungal cells from adverse environmental conditions (Durán and Nombela, 2004). The compact network of the fungal cell wall not only is essential for cell morphology and viability but also, through the activity of several adhesin proteins, mediates both intercellular adhesion and the adhesion between cells and environmental material surfaces, which is an initial and essential step in biofilm development (Finkel and Mitchell, 2011; Desai and Mitchell, 2015). In addition, components of the fungal cell wall are important for the pathogenesis of and immune response to *C. albicans* (Hoyer et al., 1999; Chaffin, 2008; Plaine et al., 2008; Lenardon et al., 2010; Hall and Gow, 2013). Thus, the fungal cell wall and membrane are excellent targets for antifungal compounds, and several clinical antifungal drugs targeting the cell wall or cell membrane have been developed (Robbins et al., 2016). However, the limited number of current therapeutic options for fungal infections and the potential adverse drug reactions remain challenges. Furthermore, human fungal pathogens are more closely related to the host than are human bacterial pathogens, so the development of new antifungals is more time consuming than the development of antibacterial drugs. These factors suggest that chitosan is a new and highly promising molecule for the treatment of human fungal infections if used in combination with an antifungal drug or a promising treatment in the clinical therapy, particular those of skin and mucosal infections. Indeed, the antifungal activity mediated by chitosan is enhanced after photodynamic inactivation (PDI) treatment (Lin et al., 2018). A global transcriptomic approach to study how *C. albicans* responds to chitosan is under investigation in the laboratory, and we believe that this genetic information will provide more insights for our development of treatments to more specifically control fungal infections in the future.

## Author Contributions

Y-TL and C-HL conceived this study and designed the experiments. P-YS, Y-TL, Y-KT, and C-HL drafted the manuscript. C-HL revised the manuscript. Y-TL and F-SD performed the mutant library screening test. P-YS and Y-KT created knockout strains and performed sensitivity tests and qPCR. P-YS and Y-TL performed the TEM. All authors read and approved the final manuscript.

## Conflict of Interest Statement

The authors declare that the research was conducted in the absence of any commercial or financial relationships that could be construed as a potential conflict of interest.

## Footnotes

1 http://www.fgsc.net/candida/FGSCcandidaresources.htm
