# Supplementary figures and images for: A Potential Antifungal Effect of Chitosan Against Candida albicans Is Mediated via the Inhibition of SAGA Complex Component Expression and the Subsequent Alteration of Cell Surface Integrity
Source: Front Microbiol. 2019 Mar 26;10:602. doi: 10.3389/fmicb.2019.00602 (PMC6443709; doi:10.3389/fmicb.2019.00602)

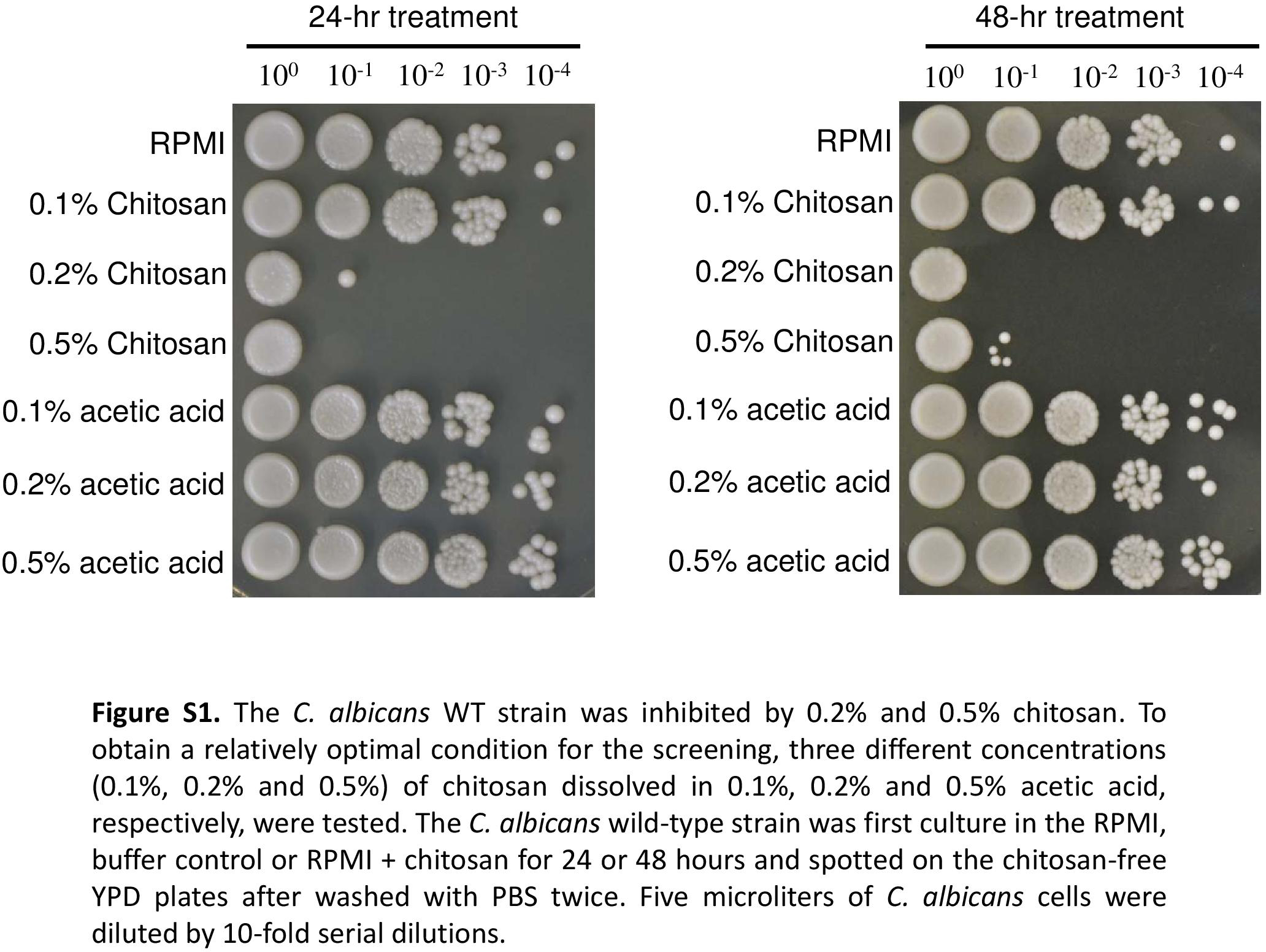

Supplement: Supplementary file 1 [file Image_1.JPEG]

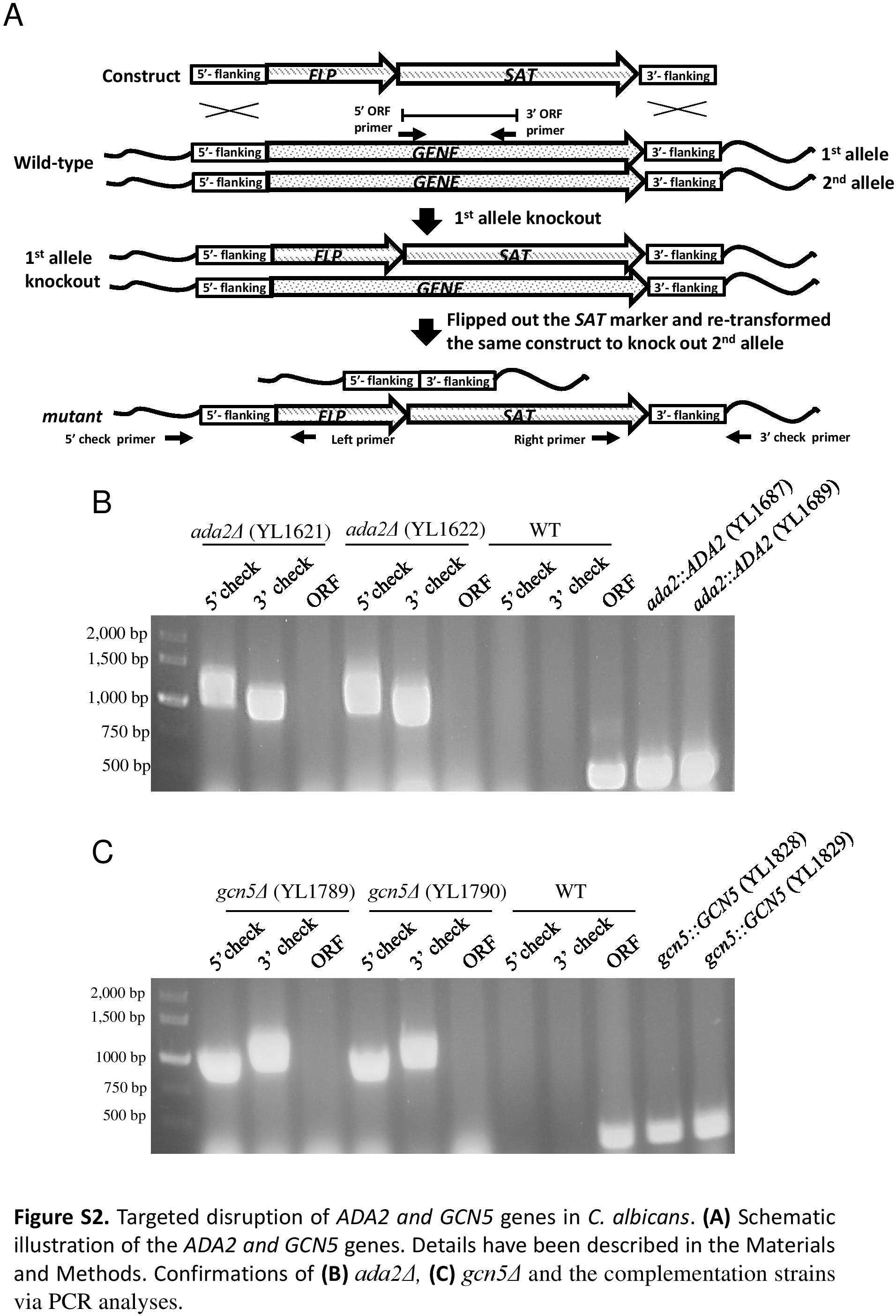

Supplement: Supplementary file 2 [file Image_2.JPEG]
