# Supplementary material for: A Potential Antifungal Effect of Chitosan Against Candida albicans Is Mediated via the Inhibition of SAGA Complex Component Expression and the Subsequent Alteration of Cell Surface Integrity
Source: Front Microbiol. 2019 Mar 26;10:602. doi: 10.3389/fmicb.2019.00602 (PMC6443709; doi:10.3389/fmicb.2019.00602)
Supplement: Supplementary file 3 [file Table_1.DOCX]

**Table S1** Strains used in this study.

| Strain name | *MTL* type | Genotype | Source |
| --- | --- | --- | --- |
| YL 2 | **a**/α | Wild type | ( Bennettand Johnson, 2006) |
| YL 1687 | **a**/α | *ada2/ada2::ADA2* | This study |
| YL 1689 | **a**/α | *ada2/ada2::ADA2* | This study |
| YL 1693 | **a**/α | *ada2/ada2* | This study |
| YL 1694 | **a**/α | *ada2/ada2* | This study |
| YL1789 | **a**/α | *gcn5/gcn5* | This study |
| YL1790 | **a**/α | *gcn5/gcn5* | This study |
| YL1828 | **a**/α | *gcn5/gcn5::GCN5* | This study |
| YL1829 | **a**/α | *gcn5/gcn5::GCN5* | This study |
| Mutant library strains* | **a**/α | *-----------------------* | (Davis et al., 2002; Nobile and Mitchell, 2005; Richard et al., 2005; Norice et al., 2007; Rauceo et al., 2008) |

*Details of the mutant library strains are listed in the supplementary Table S3.

Bennett, R. J., and Johnson, A. D. (2006). The role of nutrient regulation and the Gpa2 protein in the mating pheromone response of *C. albicans*. *Mol. Microbiol. 62*, 100-119. doi:[10.1111/j.1365-2958.2006.05367.x](https://doi.org/10.1111/j.1365-2958.2006.05367.x).

Richard, M. L., Nobile, C. J., Bruno, V. M., and Mitchell, A. P. (2005). *Candida albicans* biofilm-defective mutants. *Eukaryot*. *Cell* 4**,** 1493-1502. doi: 10.1128/EC.4.8.1493-1502.2005.

Nobile, C. J., & Mitchell, A. P. (2005). Regulation of cell-surface genes and biofilm formation by the *C. albicans* transcription factor Bcr1p. *Curr*. *Biol*. 15**,** 1150-1155. doi: 10.1016/j.cub.2005.05.047.

Norice, C. T., Smith, F. J., Jr., Solis, N., Filler, S. G., and Mitchell, A.P. (2007). Requirement for *Candida albicans* Sun41 in biofilm formation and virulence. *Eukaryot*. *Cell*. 6**,** 2046-2055. doi: 10.1128/EC.00314-07.

Rauceo, J. M., Blankenship, J. R., Fanning, S., Hamaker, J. J., Deneault, J. S., Smith, F. J., Nantel, A., and Mitchell, A. P. (2008). Regulation of the *Candida albicans* cell wall damage response by transcription factor Sko1 and PAS kinase Psk1. *Mol*. *Biol*. *Cell* 19**,** 2741-2751. doi: 10.1091/mbc.E08-02-0191.

Davis, D. A., Bruno, V. M., Loza, L., Filler, S. G., and Mitchell, A. P. (2002). *Candida albicans* Mds3p, a conserved regulator of pH responses and virulence identified through insertional mutagenesis. *Genetics* 162**,** 1573-1581.
