# Supplementary material for: A Potential Antifungal Effect of Chitosan Against Candida albicans Is Mediated via the Inhibition of SAGA Complex Component Expression and the Subsequent Alteration of Cell Surface Integrity
Source: Front Microbiol. 2019 Mar 26;10:602. doi: 10.3389/fmicb.2019.00602 (PMC6443709; doi:10.3389/fmicb.2019.00602)
Supplement: Supplementary file 4 [file Table_2.DOCX]

**Table S2** Primers used in this study.

| Primer number | Sequence (5’ to 3’) |
| --- | --- |
| 6 | CTCAACCATAGCAATCATGG |
| 7 | GCGAAAAAGTGGGCACTAAG |
| 541 | TGGACTTGTGTTGTTATCTGGACT |
| 542 | CTTGCTGTGTTTGTGTTTGTGTTG |
| 1017 | TCTGTGTATACTCAATCCAGGTGCG |
| 1018 | ACGCTACATGGTTCAAGAAACCCCC |
| 1019 | GCCTGGTAGATTGGAGTTTGACCAT |
| 1020 | TGGGAATGTCTTCCAGAGCCTGTAC |
| 1021 | GGAGCGGGGCCCAAAACTGCATATAATTGAGTCA |
| 1022 | GGAGCGCTCGAGTATGGAGTAGAGACTTGTTTAC |
| 1023 | GGAGCGCCGCGGTTC TCTTGTTGTATCAAATCAT |
| 1024 | GGAGCGGAGCTCGTC TACCCTTACTACAACTACT |
| 1085 | GTGATTGGGGAGCAGATGAA |
| 1086 | CAGCCCAATTACCCAATCCA |
| 1087 | TTGGAGTTTGGGTGCTGTTT |
| 1088 | CCATCTCCAACTGGCTTTGA |
| 1089 | TCACTTTTCGAAGCCAGACC |
| 1090 | AATACTGGCCAAGGCATCAG |
| 1147 | GGAGCGGCGGCCGCATTTATTGACAGAATTGAAA |
| 1148 | GGAGCGCCGCGGATATGCACAAAGAATTCATAGA |
| 1149 | GGAGCGGGTACCATTGAAACAGTTTGAACTGGAA |
| 1150 | GGAGCGGGGCCCATTATATAAATCTATGCAAAAC |
| 1228 | AAATGTCCCCGCTGGTTATC |
| 1229 | GGAACTACCAACACAGGCAT |
| 1234 | TGCTGGTGTTTTATCCAAACG |
| 1235 | ACTAGTGGCACGTTTAAGCA |
| 1238 | CGACGCCGAATCAAAAAGAC |
| 1239 | TATCCTCATCCGGTCCTGTC |
| 1251 | GGAGCGGGGCCCAATGAGGTTCGGTCCAGATGAA |
| 1252 | GGAGCGCTCGAGACGTGACCTTAATAGAGGAGGGC |
| 1253 | GGAGCGCCGCGGCATAAGACAAACCACGCGGG |
| 1254 | GGAGCGGAGCTCTCATGTTTGTGGTCTCTTGAGGC |
| 1255 | CGTCCATTTAACAACCGTGGA |
| 1256 | TGCCTCTGCTAATCCAGGGAT |
| 1257 | TGCATGGCAATCACAACGTATT |
| 1258 | AGAGCAAGTGTGTCCATCTGGC |
| 1493 | AGGGCAACATCTCCAATCAAAT |
| 1494 | GCATCAATGTACCACCTTCGTA |
| 1558  1559  1560  1561 | GGAGT TCCAT TAAGA GCATA TAC  ACATC CAACA GACCA CATAT  TCATT AGATA GAACC GTTAG AGT  GAGAA GGCGA CAGAG TAG |
