# Supplementary material for: A Potential Antifungal Effect of Chitosan Against Candida albicans Is Mediated via the Inhibition of SAGA Complex Component Expression and the Subsequent Alteration of Cell Surface Integrity
Source: Front Microbiol. 2019 Mar 26;10:602. doi: 10.3389/fmicb.2019.00602 (PMC6443709; doi:10.3389/fmicb.2019.00602)
Supplement: Supplementary file 5 [file Table_3.DOCX]

**Table S3** The Cq values of *ACT1* gene in each biological experiment.

Figure 6A

| **Gene** | **Sample (treatment)** | **Cq (Biological repeat)** | | |
| --- | --- | --- | --- | --- |
|  |  | **Mean Cq (1)** | **Mean Cq (2)** | **Mean Cq (3)** |
| *ACT1* | WT (RPMI, 20 min) | 18.23 | 18.05 | 19.15 |
| *ACT1* | WT (0.2% acetic acid, 20 min) | 18.45 | 18.38 | 19.07 |
| *ACT1* | WT (0.5% acetic acid, 20 min) | 19.06 | 18.36 | 19.6 |

Figure 6B

| **Gene** | **Sample (treatment)** | **Cq (Biological repeat)** | | |
| --- | --- | --- | --- | --- |
|  |  | **Mean Cq (1)** | **Mean Cq (2)** | **Mean Cq (3)** |
| *ACT1* | WT (RPMI, 20 min) | 18.39 | 18.11 | 17.25 |
| *ACT1* | WT (0.2% chitosan, 20 min) | 17.88 | 18.45 | 17.35 |
| *ACT1* | WT (RPMI, 1 hr) | 19.26 | 18.3 | 18.05 |
| *ACT1* | WT (0.2% chitosan, 1 hr) | 19.23 | 18.19 | 18.16 |

Figure 7A

| **Gene** | **Sample (treatment)** | **Cq Biological repeat** | | |
| --- | --- | --- | --- | --- |
|  |  | **Mean Cq (1)** | **Mean Cq (2)** | **Mean Cq (3)** |
| *ACT1* | WT (RPMI, 20 min) | 19.06 | 18.61 | 19.08 |
| *ACT1* | *ada2Δ* (RPMI, 20 min) | 19.07 | 19.08 | 19.49 |

Figure 7B

| **Gene** | **Sample (treatment)** | **Cq (Biological repeat)** | | |
| --- | --- | --- | --- | --- |
|  |  | **Mean Cq (1)** | **Mean Cq (2)** | **Mean Cq (3)** |
| *ACT1* | WT (RPMI, 20 min) | 19.69 | 20.26 | 20.19 |
| *ACT1* | WT (0.2% chitosan, 20 min) | 19.35 | 20.09 | 20.11 |

Figure 8A

| **Gene** | **Sample (treatment)** | **Cq (Biological repeat)** | | |
| --- | --- | --- | --- | --- |
|  |  | **Mean Cq (1)** | **Mean Cq (2)** | **Mean Cq (3)** |
| *ACT1* | SC5314 (RPMI, 20 min) | 18.20 | 18.09 | 18.12 |
| *ACT1* | *ada2Δ* (RPMI, 20 min) | 17.66 | 18.54 | 18.43 |

Figure 8B

| **Gene** | **Sample (treatment)** | **Cq (Biological repeat)** | | |
| --- | --- | --- | --- | --- |
|  |  | **Mean Cq (1)** | **Mean Cq (2)** | **Mean Cq (3)** |
| *ACT1* | SC5314 (RPMI, 20 min) | 18.39 | 18.11 | 17.25 |
| *ACT1* | SC5314 (0.2% chitosan, 20 min) | 17.88 | 18.45 | 17.35 |

Figure 9D

| **Gene** | **Sample (treatment)** | **Cq (Biological repeat)** | | |
| --- | --- | --- | --- | --- |
|  |  | **Mean Cq (1)** | **Mean Cq (2)** | **Mean Cq (3)** |
| *ACT1* | SC5314 (RPMI, 20 min) | 18.59 | 18.3 | 18.0 |
| *ACT1* | SC5314 (0.2% chitosan, 20 min) | 18.78 | 18.38 | 17.81 |
